# Mandala-inspired representation of the turbulent energy cascade

**DOI:** 10.1103/PhysRevFluids.3.100505

**Published:** 2018-10

**Authors:** Maxime Bassenne, Hyunji Jane Bae, Adrián Lozano-Durán

**Affiliations:** Center for Turbulence Research, Stanford University, Stanford, California 94305-3024, USA

Understanding the cascading process of turbulent kinetic energy from large-scale fluid motions to small-scale and lesser-scale fluid motions is critical to modeling strategies for geophysical and industrial flows. The phenomenological explanation of the transfer of energy across scales was introduced in the classical paper by Kolmogorov [1], but the concept of a turbulent cascade in terms of interactions among eddies was proposed earlier by Richardson [2] and later by Obukhov [3]. Since then, many detailed investigations, mostly in isotropic turbulence, have greatly advanced our understanding of high-Reynolds-number turbulent flows.

Attempts to unravel the mechanisms behind the cascade have relied on varying but complementary physical rationales. A classic explanation is given in terms of vortex stretching acting across scales [4,5]. Other approaches have directly tested the original idea of Richardson [2] in terms of eddy breakdown [6,7] or multifractal models for the statistical properties of the energy transfer [8]. Notwithstanding the efforts, the cascading process remains one of the most challenging problems in turbulence due to its multiscale nature. The advent of wavelet methods has opened new avenues for analyzing nonlinear processes in turbulence [9,10], partly due to their ability to unfold signals into both space and scale, simultaneously.

In this work, the continuous wavelet transform is applied to one-dimensional instantaneous velocity signals obtained from direct numerical simulation of three-dimensional isotropic turbulence. Details about the data can be found in [7,11]. The algorithm uses the complex-valued Morlet wavelet [12], converting one-dimensional physical-space velocity signals into two-dimensional arrays of wavelet coefficients that represent the local velocity fluctuations at a given scale around a fixed position. The complex wavelet coefficients, of which only the real parts are visualized in the present analysis, are subsequently mapped into polar coordinates. The radial and azimuthal coordinates represent inverse scale and position, respectively.

Figure 1 shows the resulting polar plots. They are visual evidence of the Richardson turbulent energy cascade. In particular, the pitchfork pattern that describes the distribution of energy across scales around a fixed position (moving from the center to the edge at a fixed azimuthal angle) reveals its fractal character [13]. The effect of the Reynolds number is highlighted in Figs. 1(a)–1(d). The energy-containing eddies represented in the core of the figures at small-radius values exhibit little sensitivity to the increase in Reynolds number. On the contrary, the inertial-range scales shown at larger-radius values contain an increasing number of cascading stages, visually demonstrating the larger-scale separation between the energy-containing and the dissipation ranges. The turbulent structures are shown for the largest Reynolds number in Fig. 1(e), which is a complementary version of Fig. 1(d).

## Figures and Tables

**FIG. 1.**
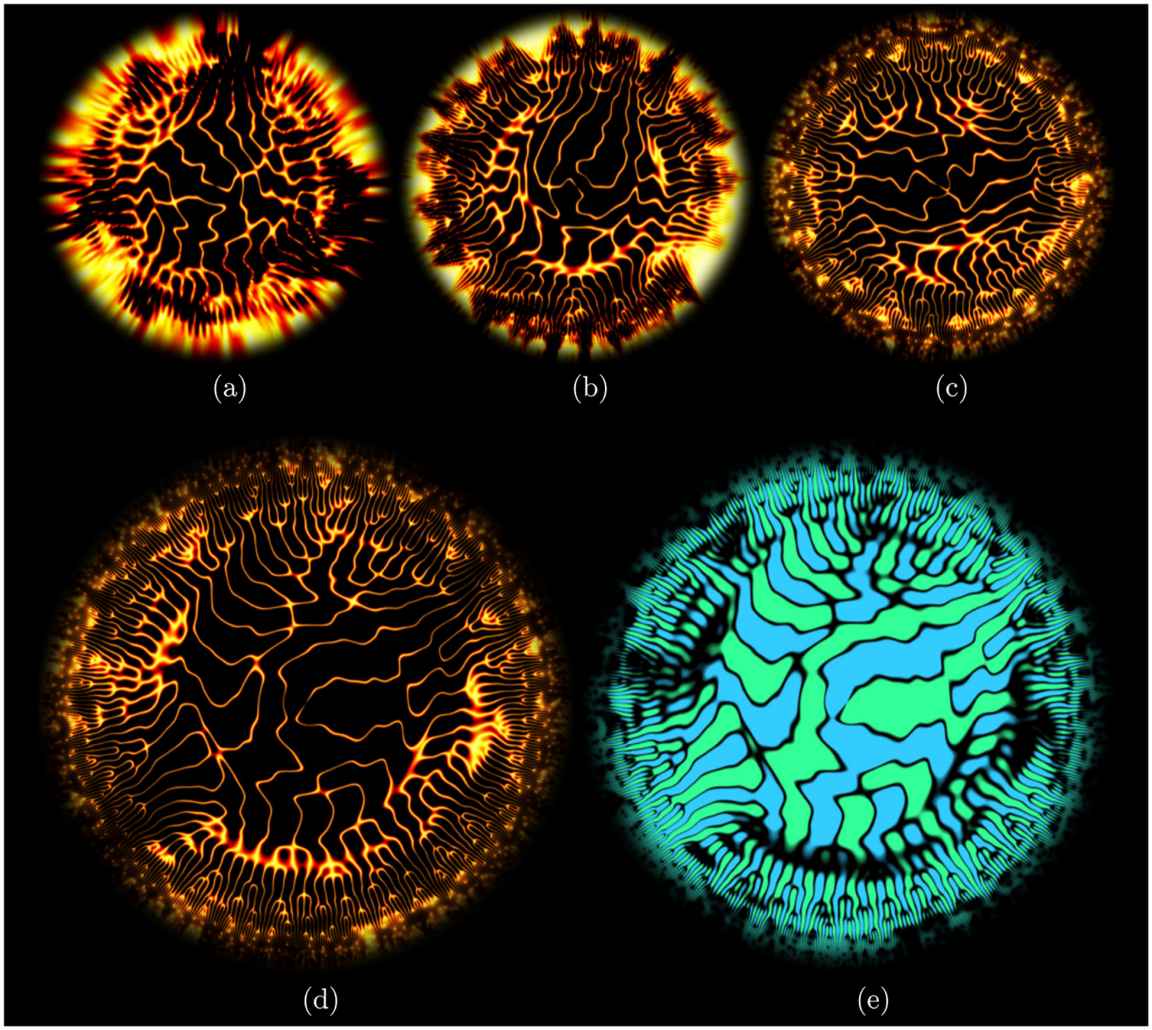
Polar plots of the wavelet transform of one-dimensional velocity signals extracted from three-dimensional isotropic turbulence data at different Taylor-microscale Reynolds number: (a) Re_*λ*_ = 140, (b) Re_*λ*_ = 380, (c) Re_*λ*_ = 1100, and (d) and (e) Re_*λ*_ = 2300. In each panel, wavelet coefficients are normalized by the scale-dependent extremum. In (a)–(d) colors (orange) highlight zero wavelet coefficients. In (e) colors represent positive (blue) and negative (green) wavelet coefficients, descriptive of turbulent structures.
